# Differential attainment and recruitment to Intensive Care Medicine Training in the UK, 2018–2020

**DOI:** 10.1186/s12909-022-03732-w

**Published:** 2022-09-12

**Authors:** Ascanio Tridente, Jack Parry-Jones, Shashi Chandrashekaraiah, Daniele Bryden

**Affiliations:** 1 Intensive Care Unit, Whiston Hospital, St Helens and Knowsley Teaching Hospitals, Warrington Road, Prescot, L35 5DR UK; 2 Intensive Care Unit, University Hospital of Wales, Heath Park Way, Cardiff, CF1 4XW UK; 3 Intensive Care Unit, Lancashire Teaching Hospitals, Royal Preston Hospital, Preston Road, Chorley, PR7 1PP UK; 4 Intensive Care Unit, Sheffield Teaching Hospitals NHS Trust, Herries Road, Sheffield, S5 7AU UK

**Keywords:** Differential Attainment, Intensive Care Medicine Training, Discrimination, Bias, Unconscious Bias, International Medical Graduate

## Abstract

**Background:**

Differences exist among doctors in examination performance, clinical and academic career progression, and prevalence of performance assessment by professional regulatory bodies. Some of these differences have been reported in relation to individual characteristics. The purpose of this study is to establish whether any specific individual characteristics are associated with performance in selection for entry into specialty training in Intensive Care in the United Kingdom.

**Methods:**

We evaluated data of 509 candidates from the national recruitment rounds of 2018/19 and 2019/20. The outcome evaluated was “success at interview". Variables reaching statistical significance at univariate logistic regression analysis were fed in the multivariable analysis to identify independent predictors of success, with additional exploratory analyses performed, where indicated.

**Results:**

The candidates’ median age was 31.5 (interquartile range, IQR 30–33.7) years, 324 (63.7%) were male, 256 (50.3%) not married/in civil partnership, 6 (1.2%) pregnant. The majority (316, 62.1%) were White British, 99 (19.5%) of Asian background, other ethnicities represented less than 20% of the sample. Of the 509 candidates, 155 (30.5%) were Atheist, 140 (27.5%) Christian; most were heterosexual (440, 86.4%); 432 (84.9%) reported no disability, while 4 (0.8%) had a minor and 1 (0.2%) had a major disability; 432 (84.9%) candidates held a UK medical degree; 77 (15.1%) a non-UK degree. At univariate logistic regression analysis (LRA) multiple factors were found to be associated with a lower likelihood of success, the strongest being an international medical graduate (IMG, holding a non-UK medical degree); others were increasing age, male gender, being married, Asian or mixed ethnicity, specific religious beliefs (Buddhism, Islam and Hinduism).

After feeding all factors significant at univariate analysis, the only two retained as independent predictors at multivariable regression were Asian ethnicity and holding a non-UK degree. Asian UK graduates success rate was 92.7%, comparable to the national average of 92.3%, the Asian IMGs success rate was significantly lower, at 45.5%.

**Conclusions:**

As the imbalances seen within the candidates of Asian background are explained by considering the country of primary medical training, the variations in performance is likely to reflect differences in training systems and understanding of the UK NHS.

## Background

Differences exist among doctors in examination performance, clinical and academic career progression, and prevalence of performance assessment by professional regulatory bodies. Some of these differences have been reported in relation to individual characteristics: gender, ethnic background and country of qualification have all been linked to outcomes [1–4]. Different explanations have been offered for the findings, including unconscious bias, potential or actual discrimination, differences in access to training, career development opportunities, experience of working in the NHS and variations in medical training between countries.

Entry to the National higher medical training programme for Intensive Care Medicine (ICM) in the UK is administered by Health Education England (HEE) West Midlands on behalf of the 4 UK nations and the Faculty of Intensive Care Medicine (FICM); the professional body responsible for the training, assessment, practice and continuing professional development of UK ICM doctors. At the time of data collected for this study, entry into the training programme follows a central selection process consisting of a written application stage, followed by an in person interview process involving 4 face to face assessment panels and one written test station. Training positions are advertised by the faculty on the recruitment pages of its website and via social media.

This current study aims to establish whether differences in baseline characteristics and demographics were related to successful performance in the selection process for entry into specialty training in Intensive Care in the United Kingdom. Data pertaining to recruitment in the years 2018/19 and 2019/20 were examined. Data beyond 2020 was not available as due to the Covid-19 pandemic the 2020/21 and 2021/22 recruitment rounds were not run as usual. The analyses described here focus on candidates’ characteristics and how they may impact performance; evaluation of the selection process itself is outside of the scope of the current study.

## Methods

### Database

We evaluated data of 598 candidates from the national recruitment rounds of 2018/19 and 2019/20. Of the 598 records available, 89 were removed from analysis because the candidates withdrew their application before or after the interview, and no score for decision on appointment (the outcome of interest) was available. The resulting database included 509 candidates. The outcome variable “success at interview” was defined as a total score at interview of 194 or above, failure was defined as a score below 194. The score of 194 was predefined by an expert Delphi panel as representing the minimum appointable score and has been used as the cut off appointable score since 2011 when the current recruitment process was developed.

Descriptive statistical analyses were performed on the baseline characteristics and the outcome variables; inferential statistical analyses were conducted using univariate and multivariable logistic regression with the outcome variable being success for the purpose of appointment. Variables reaching statistical significance at univariate analyses (pre-defined cut off *p* = 0.05) were fed in a multivariable logistic regression analysis model, to identify independent predictors of success. Missing values were handled as such and highlighted throughout the manuscript and in the tables. No attempts were made to replace them or input them in any alternative way. Multi-collinearity was evaluated using variance inflation factors. Additional exploratory analyses were performed to investigate further aspects of the recruitment process. Analyses were conducted using Stata 15.1 (StataCorp, 4905 Lakeway Drive, College Station, Texas 77,845 USA, http://www.stata.com).

Scores were available for 509 candidates (range 122–365, mean 263.6, median 271 interquartile range 143–342); the scores were normally distributed (skewness -0.74, kurtosis 3.3).

## Results

### Baseline characteristics

Baseline demographic and other characteristics for the 509 candidates are reported in Table 1. The mean age was 32.4 (SD, standard deviation 3.4) years, while the median age was 31.5 (IQR, interquartile range 30–33.7) years. The candidates were divided into four age groups: 25–30 (131 candidates, 25.7%), 30–35 (295, 58%), 35–40 (67, 13.2%) and over 40 years old (16, 3.1%), as per Table 1, which reports all baseline characteristics of the candidates’ population. There was a significant male preponderance: 324 candidates were male, representing 63.7% of the sample, whereas 171 were female (33.6%), 8 candidates preferred not to specify their gender (1.6%) and for 6 candidates gender data were missing (1.2%). Available marital status data indicated that just over half of the sample (256 individuals, 50.3%) were not married nor in a civil partnership, while 170 (33.4%) were married or in civil partnership, 22 (4.3%) preferred not to specify, and for 61 (12%) data was missing. In the whole sample only 6 individuals representing 1.2% were pregnant and 8 (1.6%) preferred not to specify whether they were pregnant or not.

**Table 1:** Candidates’ baseline characteristics

**Characteristics**	**N or Median**^a^	**% or IQR**^b^
Age group (years)		
25–30	131	25.7
30–35	295	58
35–40	67	13.2
> 40	16	3.1
All	31.5^a^	30–33.7^b^
**Gender**		
Male	324	63.7
Female	171	33.6
Not disclosed	8	1.6
Missing	6	1.2
**Marital status**		
Not married nor in a civil partnership	256	50.3
Married or in a civil partnership	170	33.4
Not disclosed	22	4.3
Missing	61	12
**Pregnancy status**		
Not pregnant	283	55.6
Pregnant	6	1.2
Not disclosed	8	1.6
Missing	212	41.7
**Ethnic origin**		
White – British, Irish, other	316	62.1
Asian, Asian British, other^c^	99	19.5
Chinese	16	3.1
Black or Black British, other^d^	11	2.2
Any other ethnic group or mixed group	42	8.3
Not disclosed	25	4.9
**Religious belief**		
Atheism	155	30.5
Christianity	140	27.5
Islam	55	10.8
Hinduism	38	7.5
Buddhism	13	2.6
Sikhism	4	0.8
Judaism	4	0.8
Other	17	3.3
Not disclosed	73	14.3
Missing	10	2
**Sexual Orientation**		
Heterosexual	440	86.4
Gay	11	2.2
Bisexual	2	0.4
Lesbian	6	1.2
Not disclosed	50	9.8
**Disability**		
None	432	84.9
Yes, limited a little	4	0.8
Yes, limited a lot	1	0.2
Not disclosed	12	2.4
Missing	60	11.8
**Country of Degree**		
UK	432	84.9
Non-UK	77	15.1

*N* Number of observations, *%* Percentage; ^a^median and ^b^interquartile range (IQR) are shown instead of count and % where relevant

^c^includes Indian, Pakistani, Bangladeshi and any other Asian background

^d^includes African, Caribbean and any other black background

Ethnic origin was varied, with 316 candidates being of White British, Irish, or from other White background (62.1% of the sample), 99 (19.5%) candidates were Asian, Asian British, Indian, Pakistani, Bangladeshi or from other Asian background, 16 (3.1%) were of Chinese origin, 11 (2.2%) were Black, Black British, African, Caribbean or from other Black background, 42 (8.3%) of any other ethnic group or of mixed background, 25 (4.9%) candidates did not specify their ethnic origin.

Of the 509 candidates, 155 (30.5%) were Atheist representing the largest subgroup, 140 (27.5%) were Christian, 55 (10.8%) were of Islamic faith, 38 (7.5%) Hindu, 13 (2.6%) Buddhist, 4 (0.8%) Sikh, 4 (0.8%) were of Judaic faith, 73 (14.3%) did not wish to disclose, 17 (3.3%) had other faith, 10 (2%) had missing data for this field.

The 509 candidates described themselves as heterosexual in the large majority of cases (440, 86.4%), with smaller percentages in the Gay (11, 2.2%), Bisexual (2, 0.4%), Lesbian (6, 1.2%) categories; 50 (9.8%) candidates did not disclose their sexual orientation. No disability was reported by 432 candidates (84.9%), data was missing for 60 (11.8%) candidates, a minor degree of disability (answer “Yes, limited a little”) was reported by 4 (0.8%) of the candidate while a significant disability (“Yes, limited a lot”) was reported by one 1 (0.2%) candidate only; disability status was not disclosed in 12 cases (2.4%).

Primary medical degree was obtained in a UK University in 432 (84.9%) cases, with 77 (15.1%) being international medical graduates (IMGs), holding non-UK primary medical degrees.

### Outcome (success at interview)

Success rates at interview are reported in Table 2. The likelihood of being offered a training position at interview varied across the age groups: the younger candidates (25–30 years old) had a success rate of 98.5%, followed by progressively lower rates for the older age groups (92.5% for 30–35 years old, 86.6% and 62.5%, for the 35–40 years old and those over 40 years of age). Candidates who were not married or in a civil partnership had a 96.5% success rate, while those married or in a civil partnership had an 85.3% success rate. Where marital status was not disclosed or missing the success rate was 100% and 91.8%, respectively. The 6 of 171 female candidates who stated that they were pregnant and those who preferred not to specify pregnancy status had 100% success rate, while the success rate amongst individuals who were not pregnant was 91.9%.

**Table 2:** Success rates across categories stratified within the various candidates’ characteristics

Characteristics	Total in category	N Successful	%
**Age group (years)**			
25–30	131	129	98.5
30–35	295	273	92.5
35–40	67	58	86.6
> 40	16	10	62.5
All	509	470	92.3
**Gender**			
Male	324	292	90.1
Female	171	164	95.9
Not disclosed	8	8	100
Missing	6	6	100
**Marital status**			
Not married nor in a civil partnership	256	247	96.5
Married or in a civil partnership	170	145	85.3
Not disclosed	22	22	100
Missing	61	56	91.8%
**Pregnancy status**^c^			
Not pregnant	165	158	95.7
Pregnant	6	6	100
Not disclosed	8	8	100
**Ethnic origin**			
White – British, Irish, other	316	313	99.1
Asian or Asian British, other^a^	99	71	71.7
Chinese	16	16	100
Black or Black British, other^b^	11	10	90.1
Any other ethnic group/mixed group	42	37	88.1
Not disclosed	25	23	92
**Religious belief**			
Atheism	155	153	98.7
Christianity	140	136	97.1
Islam	55	33	60
Hinduism	38	30	78.9
Buddhism	31	11	84.6
Sikhism	4	4	100
Judaism	4	4	110
Other	17	17	100
Missing	10	10	100
Not disclosed	73	72	98.6
**Sexual Orientation**			
Heterosexual	440	405	92
Gay	11	11	100
Bisexual	2	2	100
Lesbian	6	6	100
Not disclosed	50	46	92
**Disability**^c^			
None	432	396	91.7
Yes, limited a little	4	4	100
Yes, limited a lot	1	1	100
Not disclosed	12	12	100
Missing	60	57	95
**Country of Degree**			
UK	432	425	98.4
Non-UK	77	45	58.4

N (%) Successful, number (percentage) of successful candidates within category

^a^includes Indian, Pakistani, Bangladeshi and any other Asian background

^b^includes African, Caribbean and any other black background

^c^The categories “pregnancy” and “disability” contain rare events. The results from analysing these variables are likely to be underpowered, hence the absence of a detectable association may be simply due to lack of sufficient power

Success rates varied significantly across the various ethnicity groups: candidates of White background were successful in 99.1% of cases, while success rate was 71.7% for candidates of Asian background and 90.1% for Black background candidates. The success rate was 100% in the Chinese group, 88.1% in any other ethnic and mixed group and 92% for those whose ethnic origin was not stated.

Candidates who stated they were Atheist had a 98.7% success rate, and the rate was 98.6% for those not wishing to disclose their religious affiliation. Success rates were 97.1% for those who declared to be of Christian faith, 84.6% for those who followed Buddhism, 60% and 78.9% in case of Islamism and Hinduism, respectively. The success rate for Sikhism, Judaism, or in case of missing data or other belief was 100%.

Success rates by sexual orientation were 92% for heterosexual and those not disclosing, while for Gay, Bisexual and Lesbian the success rate was 100% in all cases.

For the group without disability and those where disability data was missing, the success rates were 91.7% and 95%, respectively. Candidates not wishing to disclose and those declaring any disability (whether limited or high level) had a 100% success rate.

The success rate for UK medical school graduates was 98.4% versus 58.4% for non-UK degree holders.

Pregnancy status and disability are rare events in this study. Given the small counts in those categories, the results from analyzing those variables are most likely underpowered, hence the absence of a detectable association may be simply due to lack of sufficient power.

### Inferential analyses

#### Univariate analyses

Table 3 reports the results of univariate LRAs for variables associated with statistically significant effects. The reference category for each variable is indicated in the brackets: age group (25–30 years); gender (female); married/civil partnership (not married/not in a civil partnership); ethnic origin (White-British, Irish, Other); religious belief (Christianity); country of degree (UK). Variables not displayed in Table 3 failed to reach pre-determined statistical significance and were associated with no notable effect sizes or odds ratios. At univariate logistic regression analysis (LRA) performed for potential predictors of outcome, age was a significant factor associated with success. Increasing age was associated with a decrease in likelihood of success. Every increase in age group by 5 years was associated with an Odds Ratio (OR) of 0.74, 95% confidence interval (95% CI) 0.65 – 0.84, *p* < 0.001; hence for every 5 years increase in age group there was a 26% approximate decrease in the odds of success at interview. Being of male gender was also associated with a lower likelihood of success at interview (OR 0.39, 95% CI 0.17 – 0.9, *p* = 0.028) compared to baseline. Being married was also associated with lower success rate (OR 0.21, 95% CI 0.1—0.47; *p* < 0.001), as were Asian ethnic origin (OR 0.02, 95%CI 0.01–0.08; *p* < 0.001), mixed ethnicity/any other ethnicity (OR = 0.07, 95% CI 0.02–0.31; *p* < 0.001) and where the ethnic origin was not stated (OR 0.1, 95% CI 0.02- 0.7; *p* = 0.019).

**Table 3:** Univariate Logistic Regression Analyses results where statistical significance was reached (*p* < 0.05)

Characteristics	OR	95% CI	*p*
Age group (5 years increments)	0.74	0.65 – 0. 84	< 0.001
Male Gender	0.39	0.17 – 0.9	0.028
Married or in a civil partnership status	0.21	0.1 – 0.47	< 0.001
Ethnic origin			
Asian or Asian British, other^a^	0.02	0.01 – 0.08	< 0.001
Any other group or mixed group	0.07	0.02 – 0.31	< 0.001
Not disclosed	0.11	0.02 – 0.7	0.019
Religious belief			
Islam	0.04	0.01 – 0.14	< 0.001
Hinduism	0.11	0.03 – 0.39	0.001
Buddhism	0.16	0.03 – 0.98	0.048
Non-UK Degree	0.02	0.01 – 0.06	< 0.001

*OR* Odds ratio, *95% CI* 95% Confidence Interval, *p p* value

^a^includes Indian, Pakistani, Bangladeshi and any other Asian background

On univariate analysis Buddhism, Islam and Hinduism were also associated with lower likelihood of success (OR = 0.16, 95% CI 0.03 – 0.98; *p* = 0.048 for Buddhism, OR 0.04, 95% CI 0.01–0.14; *p* < 0.001 for Islam, OR 0.11, 95% CI 0.03–0.39; *p* = 0.001 for Hinduism). There were no significant differences regarding sexual orientation and disability status.

Holding a primary medical degree of non-UK origin was strongly associated with lower likelihood of success (OR 0.02, 95% CI 0.01 – 0.06, *p* < 0.001).

#### Multivariable and further analyses

After feeding all factors significant at univariate analysis, the only two factors retained as independent negative predictors at multivariable regression were Asian ethnic origin and holding a non-UK degree (Table 4).

**Table 4:** Multivariate Logistic Regression Analyses results – factors retained as independent predictors of success at interview

Characteristics	OR	95% CI	*p*
Asian Ethnic origin^a^	0.08	0.02 – 0.46	0.004
Non-UK Degree	0.07	0.02 – 0.21	< 0.001

*OR* Odds ratio, *95% CI* 95% Confidence Interval, *p p* value

^a^includes Indian, Pakistani, Bangladeshi and any other Asian background

Additional analyses were performed to explore further these findings. The IMGs’ success rate was compared to that of UK graduates within the Asian ethnic group found at disadvantage in terms of interview outcome: while Asian UK graduates success rate was 92.7%, hence comparable to the national average of 92.3%, the Asian IMGs success rate was significantly lower, at 45.5%. This finding was confirmed by LRA stratified by country of graduation: holding a degree from a country outside the UK was associated with OR of success 0.07 (95% CI 0.02- 0.21, *p* < 0.001) within the Asian subgroup of candidates and is therefore a significant explanatory factor for the apparent lower success rate for the Asian ethnic subgroup.

When comparing country of primary medical degree within different ethnic groups, the proportion of IMGs was highly variable and a higher proportion of IMGs was noted within the Asian ethnic group (Table 5).

**Table 5:** Success rates and IMG proportion across ethnicity categories

Characteristics	Total in category	N (%) Successful	N (%) IMG
**Ethnic origin**			
White – British, Irish, other	316	313 (99.1)	12 (3.8)
Asian or Asian British, other^a^	99	71 (71.7)	44 (44.4)
Chinese	16	16 (100)	5 (31.3)
Black or Black British, other^b^	11	10 (90.1)	9 (81.8)
Any other ethnic group/mixed group	42	37 (88.1)	0 (0)
Not disclosed	25	23 (92)	7 (28)

*N (%) Successful* Number (percentage) of successful candidates within category, *N (%) IMG* Number (%) of IMG within ethnic category

^a^includes Indian, Pakistani, Bangladeshi and any other Asian background

^b^includes African, Caribbean and any other black background

## Discussion

Differential attainment is defined as variation in a group’s, as opposed to an individual’s, attainment based on their protected characteristics of age, gender, disability, marriage or civil partnership, pregnancy, race, religion or belief, and sexual orientation (Equality Act 2010). For medical careers, broadly speaking, differential attainment could start at medical school entry and progress through examination success, job selection, speciality options, appointment onto speciality training, research opportunities, consultant appointments and higher national opportunities and appointments. Differential attainment has been examined by other specialities [5]. It is very important for the Faculty of Intensive Care Medicine, for our patients and for ICM as a speciality, that opportunities are open and fair to all. More than that, these opportunities also need to be perceived as, and seen to be fair by those considering the speciality as a career especially if their background is from a group which has faced discrimination.

Our study evaluated the national recruitment process for training in Intensive care Medicine for the years 2018/19 and 2019/20, examining all available baseline characteristics which may be associated with success at interview from all 509 candidates for whom the outcome of interest (success at interview defined by achieving the minimum appointable score) was available.

At univariate analysis the following factors were found to be associated with a lower likelihood of success at interview: increasing age, male gender, marriage or civil partnership, Asian or other group / mixed group ethnicities, Islamic, Hindu and Buddhist Faiths, and holding a non-UK primary medical degree. In particular, the most significant difference in success rate was based on whether the candidate had attended a UK medical school or a medical school from any other country. The success rate for UK medical school graduates and degree holders was 98.4% versus non UK degree holders (58.4%).

When all potential explanatory factors were fed into a multivariable model, the only two variables retained as independent predictors at regression analysis were Asian ethnic origin and holding a non-UK primary medical degree. The reasons which could be at the origin of these imbalances are probably related to differences in training systems. In fact, when the International medical graduates’ success rate was compared to that of UK graduates within the Asian ethnic group, we found that the Asian UK graduates success rate was 92.7%, hence comparable to the national UK average (92.3%). On the contrary, the Asian non-UK graduates’ success rate was 45.5%, well below the national average, explaining the difference in success within that ethnic group. The findings suggest that such phenomenon could be attributable to the candidates’ understanding of UK training and the intensive care interview system, and therefore their preparedness for it.

Evidence from undergraduate medical education suggests that social networks are important, and high achieving students are more likely to have at least a clinician in their social network. Interestingly, it has been reported that BAME (Black, Asian and minority ethnic) and Muslim students are less likely to have such links [6]. This means that overseas graduates may not be benefitting from the advice and support that others, who have previously made successful applications to ICM training, can provide. In order to address these imbalances, changes in how local inductions and training are conducted may need to be introduced to ensure that, with a supportive environment, opportunities for learning and workplace based experience are equally available to those with different training backgrounds. These changes, along with knowledge and understanding of the cultural differences among trainees of different backgrounds, are likely to ensure that trainers and supervisors are able to support all trainees equally in the assessment processes such as interviews and exams. These are fundamental steps and of great impact, considering that International medical graduates represent approximately 25–30% of the NHS workforce. The number of IMGs has increased over the last 5 years and over 10,000 IMGs registered with the General Medial Council (GMC) in 2020, which is more than UK qualified doctors in that same year [7]. Given that IMGs form over a quarter of the NHS medical workforce, it is important to ensure they have equal opportunities to access training, career progression and job opportunities, as several reports have suggested IMGs have been disadvantaged in some aspects of recruitment or career progression [8, 9].

For recruitment in Intensive Care Medicine, the interview stations consist of a portfolio section, one on the assessment and prioritisation of tasks, a clinical scenario relevant to UK practise and a communication skills scenario. All these tend to be areas IMGs struggle with, particularly during their initial period following arrival to the UK, hence requiring more support and guidance for IMGs to perform well in these domains [10]. Finally entry into ICM training does not require any minimum period of work and experience in the NHS, potentially leading to IMGs with minimal familiarity with the NHS applying for these training positions and being unable to demonstrate the necessary attributes at interview to achieve the minimum appointable score.

The competition for National Training Numbers (NTNs) has become more competitive with more than two candidates for each NTN. As entry becomes more competitive it is important that the process is as fair and transparent as possible.

In order to address the challenges around IMG differential attainment, the Careers Workforce and Recruitment committee of the FICM appointed an IMG project lead in 2019, to evaluate the various aspects of support and guidance for IMG doctors considering a career in ICM in the UK. Furthermore, a separate IMG section on the FICM website is aimed specifically at giving overseas doctors detailed and up to date information to help make an informed decision about moving to the UK, to pursue a career in ICM and provide a broad understanding of the working of the NHS in general and ICM in particular [11]. Under this aspect the “New to NHS” national simulation programme, developed in collaboration with the Royal College of Anaesthetists, is open to all IMG doctors new to the UK, interested in careers in ICM and Anaesthesia, to help them understand the working of the NHS, develop the relevant communications skills, and give access to simulation training on management of critical incidents [12, 13]. The FICM is also in consultation with all its Regional Advisers in ICM to facilitate the establishment of IMG leads in each Health Education England (HEE) training unit, to provide further support to IMGs about exams, interviews and career progression.

A separate consideration regards the lack of data about the number of IMGs in various locally employed (non-training) positions and associated career support or supervision available. There is an urgent need to collect such data, as effective supervision and regular feedback in non-training positions, along with enhanced induction, have been identified as a key intervention to address differential attainment [8, 14]. Moreover diversifying the interview panel has the potential to minimise the impact of bias, either conscious or unconscious. Further work is ongoing to develop opportunities for IMGs in senior educational leadership roles, such as college tutors, training programme directors, regional advisers and other senior roles at the Faculty. Any perceived bias towards IMGs can worsen career outcomes, while IMGs in leadership roles may act as models and provide motivation, which in turn are likely to improve success rates [8], while adequate mentorship and peer support can be of benefit for IMGs, particularly for those in difficulty [14].

### Potential limitations and future studies

A potential source of limitations in the current study is the fact that some of the variables have very small counts within some of the categories (pregnancy status and disability in particular). As the current analyses are based on a convenience sample of all available candidates, and within a time-window during which recruitment processes were consistent, the statistical power cannot be increased by expanding the sample. Nevertheless, authors felt that it was important to retain the essential granularity about all protected characteristics.

It may be argued that a potential additional external limitation to the study may be identified in the selection process itself. A Delphi panel methodology was used to establish a pass score. While it may be argued that such approach could lead to additional bias, based on the perception of which performance indicators may be able to predict future success, such method is commonly used in the medical field and often relied upon in developing clinical guidance, and it is unlikely to have induced significant bias in the selection process.

The results presented in this paper are based on analyses performed relying upon the available categories for the data collected (as available to the authors). For example, the notions of race, ethnicity and national origin are blended, and this fact may lead to possible confounding, as these different theoretical constructs are combined, rather than described in bespoke categories. In order to minimize the potential confounding effect of such limitations, wherever possible, the granularity of the data has been maintained throughout the analyses and in the manuscript.

Finally, the current study focusses on the applicants’ characteristics being the primary explanation of any differential attainment or bias they might experience. There may be other potential sources in the assessment process, for example within the instruments relied upon (including type and format of assessments and questions). This is an area which is outside the scope of this study and deserves further investigation.

## Conclusions

Our study evaluated the national recruitment process for training in Intensive care Medicine for the years 2018/19 and 2019/20, examining baseline characteristics potentially associated with success at interview from a total of 509 candidates. Univariate analysis identified multiple variables associated with likelihood of success at interview, but when all potential explanatory factors were fed into a multivariable model, the only two variables retained as independent predictors at regression analysis were Asian ethnic origin and holding a non-UK primary medical degree. It is likely that these imbalances in achieving the minimum appointable score are reflective of differences in training systems and understanding of the UK NHS.

While it is reassuring that our study found no evidence of disadvantage based on gender, sexual orientation, marital status, pregnancy, or disability, there are some limitations to the current analyses mainly related to the small sample size in some categories, as described in the discussion/limitations section. Furthermore, selection into specialty training is only one aspect of differential attainment in medical careers. Other areas such as consultant appointments and opportunities for senior leadership positions in ICM are subject to additional study and review by the Faculty. The UK is a multi-ethnic society, and it is likely to benefit patient outcomes if the doctors that care for them are well trained and their backgrounds reflect those of the wider society they are drawn from.

## Data Availability

Reasonable requests to access the datasets analysed will be adjudicated by the Faculty of Intensive Care Medicine (contact@ficm.ac.uk).

## Abbreviations

BAME: Black, Asian and Minority Ethnic
FICM: Faculty of Intensive Care Medicine
GMC: General Medial Council
HEE: Health Education England
ICM: Intensive Care Medicine
IMG: International medical graduate
IQR: Interquartile range
LRA: Logistic regression analysis
NHS: National Health System
NTN: National Training Numbers
OR: Odds Ratio
